# Comparative Chemical and Physical Characterization of Biomimetic Versus Commercial Hydroxyapatites for Tooth Enamel Repair

**DOI:** 10.3390/biomimetics10100672

**Published:** 2025-10-06

**Authors:** Marco Lelli, Ismaela Foltran, Rossella Pucci, Fabrizio Tarterini

**Affiliations:** 1 Department of Industrial Chemistry “Toso Montanari”, University of Bologna, 40126 Bologna, Italy; fabrizio.tarterini@unibo.it; 2 Incos-Cosmeceutica Industriale, Funo di Argelato, 40050 Bologna, Italy; ismaela.foltran@incosgroup.it (I.F.); rossella.pucci@incosgroup.it (R.P.)

**Keywords:** hydroxyapatite (HAp), enamel repair, FTIR-ATR, XRD, SEM/EDX, DLS, biomimetic apatite

## Abstract

**Background**: Substituted hydroxyapatites (HAps) are widely used in oral-care formulations for enamel repair; however, head-to-head comparisons among commercial grades remain limited. **Objective**: To compare four commercial HAps: A (Kal-HAp), B (FL-HAp), C (FL-HAp-SC), and D (microRepair^®^, a biomimetic Zn–carbonate-substituted HAp) and to evaluate their ability to form an enamel-like coating in vitro. **Methods**: We characterized the powders by X-ray diffraction (crystalline phase, Landi crystallinity index), FTIR-ATR (phosphate/carbonate bands), SEM/EDS (morphology, surface Ca/P), and DLS (particles size, ζ-potential). In vitro, human enamel sections were treated with 5% slurries in artificial saliva; surface coverage was quantified by image analysis on SEM. **Results**: All commercial materials analyzed in this work were composed of HAp. Differences were observed between HApin terms of crystallinity-range [2 Theta 8.0–60.0°], carbonate substitution (ATR [carbonate group evaluated −870 cm^−1^]), and particle size (DLS [in a range 0.1–10,000 nm], Z-mean [mV]). On enamel, all samples form a hydroxyapatite layer; coverage differed between groups ([A] 28.83 ± 7.35% vs. [B] 31.11 ± 3.12% vs. [C] 57.20 ± 33.12% vs. [D] 99.90 ± 0.12%), with the biomimetic Zn–carbonate-substituted HAp showing the highest coverage, and the post-treatment Ca/P ratio approached values similar to those of dental enamel. **Conclusions**: Complementary physic-chemical signatures (crystallinity, carbonate substitution, and morphology) relate to enamel-surface coverage in vitro, providing evidence base for selecting HAp grades for enamel-repair formulations, which is a practical implication for product design.

## 1. Introduction

Dental erosion is defined as a form of chemical wear of dental hard tissue, characterized by the absence of bacterial involvement [1]. Its clinical incidence is progressively increasing and it represents, after periodontal disease and caries, one of the main oral conditions capable of causing symptoms of functional impairment [2,3,4,5].

The etiology is multifactorial but is closely related to the marked increase in the consumption of low-pH beverages, such as soft drinks, fruit juices, and energy drinks [6,7]. Additional acid sources include syrup-form drugs, analgesics, vitamin C supplements, and environmental exposure to acidic agents in specific occupational settings [8,9,10,11].

Damage to mineralized tissue results from the erosive action of acids on the superficial layers of enamel, resulting in the demineralization and dissolution of the inorganic phase [11,12,13,14,15,16]. HAp is the predominant component of dental tissues, accounting for approximately 95% of the total weight in enamel and 75% in dentin. The determining factor for the rate of dissolution is the solubility of HAp itself, which is strictly dependent on pH; an additional protective role is attributed to the acquired salivary pellicle [17,18].

Current preventive and therapeutic approaches—most notably fluorides [19,20,21,22,23,24]—primarily inhibit apatite dissolution rather than drive true recrystallization or replacement of lost mineral. Although HAp is the fundamental determinant of the mechanical properties of dental tissues, unlike bone, enamel and dentin do not possess an intrinsic regenerative capacity: enamel is cell-free, and dentin exhibits neoapposition activity limited to the pulpal aspect. Therefore, the reconstruction of lost dental tissues can only occur through the application of alloplastic replacement materials for prosthetic purposes [19,20].

Demineralized areas and micrometer-scale defects of the enamel surface—induced by erosive processes and micro-wear [25,26,27,28,29]—are not amenable to endogenous biological self-repair nor to complete restoration with conventional prosthodontic/restorative approaches. The tooth surface is acellular and lacks the capacity to regenerate enamel or dentin lost through exposure to acidic foods and beverages. Consequently, demineralized regions cannot be biologically recovered and cannot be fully reconstituted without conventional prosthetics or restorations.

Hydroxyapatite (HAp) is a natural mineral that forms the bulk of tooth enamel and dentin. When formulated into toothpaste or mouthwash, hydroxyapatite helps repair enamel by filling microscopic defects, remineralizing tooth surfaces, reducing sensitivity, and inhibiting plaque formation.

HAp is then undoubtedly one of the best candidates for repairing tooth enamel. Several published scientific articles [30,31,32,33,34] have clearly shown that the degree of interaction between hydroxyapatite and tooth enamel depends on the chemical and physical characteristics of HAp itself. A biomimetic HAp is usually able to mimic (in terms of chemical and physical characteristics) human hydroxyapatite, thus resulting in more reactive within biological systems.

These characteristics make biomimetic hydroxyapatite particularly reactive in biological systems [35], such as the oral cavity, making it extremely similar to the dental surface, as it mimics the chemical and physical characteristics of the apatite crystals that form the tooth itself.

Conversely, HAp with chemical and physical properties different from those of the tooth is less effective at bonding and repairing the tooth surface [32,33,34].

This work will therefore investigate the chemical and physical characteristics of three commercial HAps, compared to a biomimetic HAp, specifically synthesized to best interact with the tooth enamel surface [30,36].

This study aims to elucidate how hydroxyapatite materials with distinct physicochemical properties with different degrees of biomimetism deposit on dental substrates (enamel/dentin), forming protective coatings that help maintain tooth health and structural integrity [37,38].

While the term “biomimetic HAp” is often used broadly for ion-substituted hydroxyapatite, the HAp examined here is biomimetic specifically because its substitutions (Zn^2+^, CO_3_^2−^), morphology, and crystallinity closely mirror natural dental apatite.

A HAp with these characteristics is found to be much more active within a biological system and tends to bind to the biogenic HAp of the teeth, repairing and protecting them.

## 2. Materials and Methods

### 2.1. Hydroxyapatite Used

The HAps used in this work are as follows:

- A: Kal-Hap; Italy
- B: FL-HAP; Japan
- C: FL-HAP-SC; Japan
- D: microRepair^®^, a biomimetic Zn–carbonate-substituted Hap; Italy

### 2.2. Chemical-Physical Characterization of Hydroxyapatites

#### 2.2.1. Morphological Characterization

Each HAp was characterized through SEM analysis (SEM EVO 50 EP, Carl Zeiss Inc., Cambridge, UK) for morphological characterization. Images were taken at various magnifications to evaluate the morphology and the size and distribution of the particles/crystals present.

To evaluate the elemental composition, EDX elemental analysis was performed with the EDAX Inca Energy 350 X-Max50 detector (Oxford Instruments, Abingdon, Oxfordshire, UK). The analyses were conducted at variable pressure, which was used to test the samples without having to perform a coating procedure on the surface layer that could have altered the enamel surface. To evaluate the composition, 10 images were taken at random surfaces of the top of the enamel, and the elemental composition of each was evaluated.

#### 2.2.2. X Ray Diffraction

XRD (X-ray diffraction) analysis was also performed on each HAp to evaluate its crystallinity.

The analysis was performed by analyzing a suitable quantity of powdered samples. The diffractogram was obtained considering an angle between 2 Theta 8° and 2 Theta 60°, using a Rigaku Miniflex 600R powder diffractometer—Rigaku, Akishima-shi, Japan. The analysis was performed at room temperature.

The diffractogram was analyzed using X-Pert Program (High Score Plus, Malvern, UK).

The analysis of the diffractogram leads to the determination of the crystalline phase but also to the determination of the degree of crystallinity that was obtained using the Landi method based on this formula [39]:

Xc = 1 − (V_112/300_/I_300_)

where:

V_112/300_ represents the intensity in the diffractogram of the diffraction maxima between the 112 signal (at position 2 Theta 32.19) and the 300 signal (at position 2 Theta 33.04);

I_300_ represents the intensity of the diffraction maxima of the 300 signal (at position 2 Theta 33.04)

#### 2.2.3. Infrared Analysis (ATR)

Each HAp sample was analyzed by ATR-FTIR, using the NICOLET SUMMIT X FTIR SPECTROMETER—Waltham, MA, USA with Germanium Crystal for Smart iTX/iD7/Everest—Waltham, MA, USA. The presence of the carbonate ion is determined by the observation of the intensity of the absorption signal at 870 cm^−1^. The presence of a phosphate group is observed in the absorption signal at 1030–1100 cm^−1^.

The qualitative evaluation of the absorption bands was carried out using the OMNIC Paradigm program.

#### 2.2.4. Dimensional Analysis (DLS) and ζ-Potential

The size of HAp particles was also evaluated using DLS—Dynamic Light Scattering—with a 4 mW He/Ne 632.8 nm laser source. The analysis aimed to evaluate the possible presence of nanoparticles and to evaluate the size of stable HAp particles in aqueous suspension.

The analysis was performed by suspending a suitable aliquot of the sample in aqueous suspension (demineralized water), and after an equilibration time of 120 s (time during which any excessively coarse particles have time to settle to the bottom of the cell used for the analysis), the size of the particlesremaining in the aqueous suspension was evaluated.

ζ-potential is the key parameter controlling electrostatic interactions in particle dispersions. ζ-Potential is critical for understanding dispersion stability. It is used to optimize suspension, emulsion formulations, and predict their long-term stability.

A small quantity of samples, appropriately diluted in demineralized water, was inserted into the appropriate cell. The evaluation of the particle dimensions and ζ-Potential was carried out using the Zetasized Advanced program.

### 2.3. In Vitro Test-Protocol

1. Collect 15 human teeth extracted at a trusted dental practice. According to (institutional/national) policy, this work does not constitute human subject research, as only fully de-identified extracted teeth were used.
2. Perform an initial post-extraction treatment on the teeth, leaving them to soak in a hypochlorite solution for 24 h to remove any traces of organic material still present on the tooth and to sterilize it, removing any biological material present.
3. After 24 h, rinse the teeth under running water for 2 to 3 min to remove any traces of hypochlorite solution.
4. Sectionthe teeth using a hand drill or a small vice to minimize dust generation and prevent artifacts. This step optimizes specimen preparation for the subsequent scanning electron microscopy (SEM) analysis.
5. Prepare an artificial saliva solution [40].
6. Divide the teeth into five groups of three teeth each:

Group A: group treated with Kal-Hap;

Group B: group treated with FL-HAP;

Group C: group treated with FL-HAP-SC;

Group D: group treated with microRepair^®^, a biomimetic Zn–carbonate-substituted Hap;

Group E: Reference group (immersed in only artificial saliva without the use of powder).

The groups (Groups A, B, C and D) were all created using theappropriate amount of HAp, to obtain a concentration equal to 5% HAp in the artificial saliva.

The mixture thus obtained (Slurry) was stirred until the inorganic phase was completely homogenized within the artificial saliva. It was then placed in contact with the individual portions of teeth for a total time of three minutes.

7. At the end of the contact time, rinse each tooth section under running water at room temperature for 10 s, and then leaveto air-dry until completely dry.
8. At the end of the treatment, analyze the teeth with SEM and EDX for surface evaluation. From the SEM micrographs, it is possible to observe the degree to which the deposited HAp covers the tooth enamel. Subsequently, using a specific graphics program (see Section Calculation of the Area Covered by Hydroxyapatite), it is possible to evaluate the percentage of the coverage of the tooth enamel by the deposited HAp. The objective of the in vitro evaluation is to assess the ability of different HAps to bind to the enamel surface and thus evaluate their ability to effectively repair the natural erosion it undergoes.

#### Calculation of the Area Covered by Hydroxyapatite

SEM micrographs were analyzed with image-analysis software to quantify the percentage of enamel surface covered by hydroxyapatite. Each image was initially converted into a bitmap format, and then the pixels corresponding to the deposited hydroxyapatite coating were colored. Using a specific Photoshop function, it was possible to count the number of colored pixels (corresponding to the apatite coating) and evaluate their percentage compared to the total number of pixels in the entire image.

## 3. Results

### 3.1. Powder Characterization

Initially, the nature of the crystalline phase and the degree of crystallinity was evaluated through PXRD analysis with diffractograms that were collected from 2 Theta 8.0 to 2 Theta 60.0.

**Figure 1:**
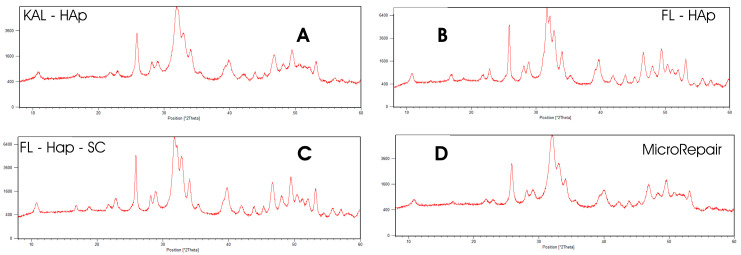
X Ray Diffraction patterns of (**A**) Kal-Hap **(B**) FL-HAp; (**C**) FL-HAp-SC; and (**D**) microRepair^®^.

Figure 1 shows that the diffraction peaks of all four samples are consistent with hydroxyapatite (HAp).

Table 1 shows the crystalline phase detected and the degree of crystallinity calculated by the reported formula (Landi method).

**Figure 2:**
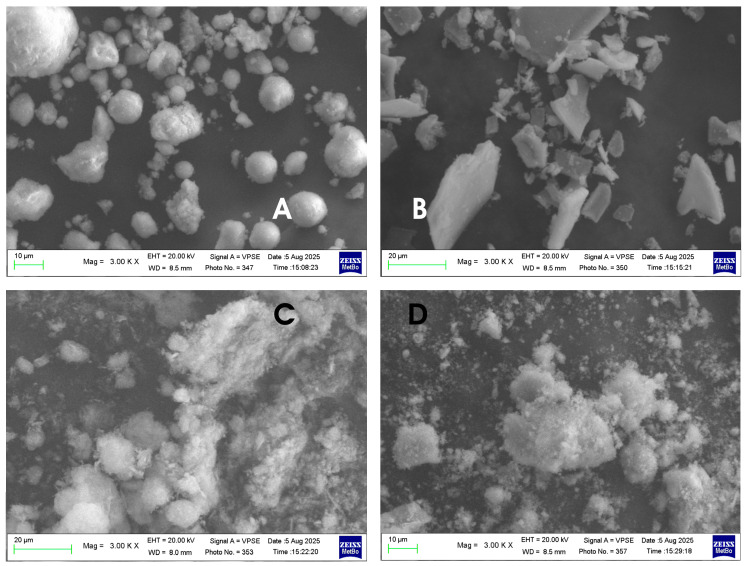
SEM micrographs of (**A**) Kal-HAp; (**B**) FL-HAp; (**C**) FL-HAp-SC; and (**D**) microRepair^®^.

Figure 2 shows the different morphologies of the hydroxyapatites analyzed.

The morphologiesof the Kal-HAp (Figure 2A) and FL-HAp (Figure 2B) samples appear to be very different from each other, while the morphologies of the samples of FL-HAp-SC (Figure 2C) and microRepair^®^ (Figure 2D), a biomimetic Zn–carbonate-substituted Hap, appear to be similar to each other, but, at the same time, completely different from the samples seen previously.

The Calcium/Phosphorus (Ca/P) ratio of all the HApsanalyzed and evaluated through the analysis carried out with the EDX probe is between 1.55 and 1.69, as shown in Table 2. All these values are similar to those typical of a HAp, whose typical Ca/P ratio is equal to 1.67.

ATR-FTIR analyses were performed to assess phosphate absorption bands and to detect the presence of carbonate within the apatitic structure [41,42].

The evaluated bands were the following:

ν (PO_4_) 1030–1100 cm^−1^;

ν (PO_4_) ~960 cm^−1^;

ν (CO_3_) ~870 cm^−1^;

ν (CO_3_) 1410–1470 cm^−1^;

OH ~3570 cm^−1^.

**Figure 3:**
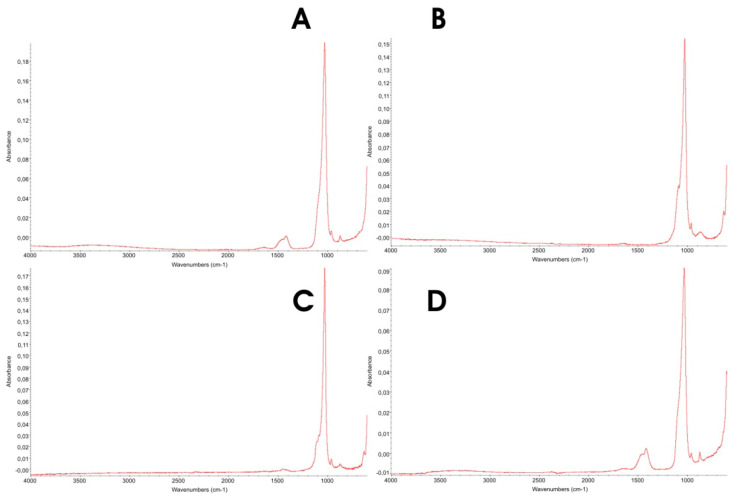
ATR spectra of (**A**) Kal-HAp; (**B**) FL-HAp; (**C**) FL-HAp-SC and (**D**) microRepair^®^, collected between 4000 cm^−1^–600 cm^−1^.

Figure 3 shows the full ATR-FTIR spectra of the four HAp samples, collected over 4000–600 cm^−1^, where the absorption bands of the relevant functional groups are evident.

**Figure 4:**
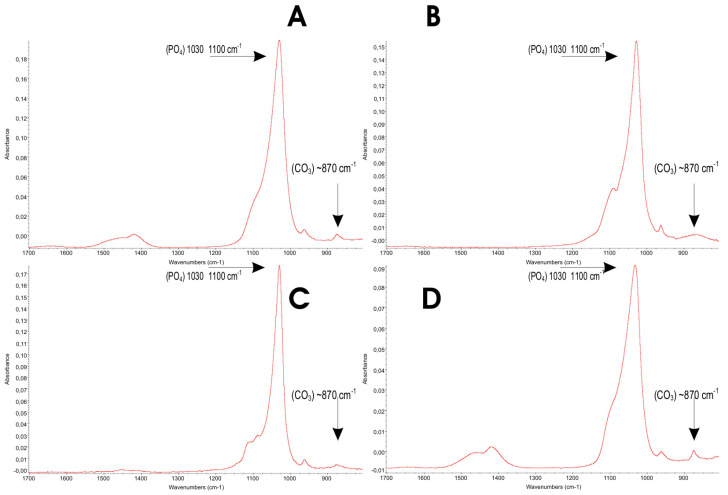
ATR spectra of (**A**) Kal-HAp; (**B**) FL-HAp; (**C**) FL-HAp-SC (**D**) and microRepair^®^; magnification between 1700 cm^−1^–700 cm^−1^ to better identify the absorption bands of interest relating to the phosphate group and the carbonate group.

Figure 4 provides an expanded view of Figure 3 over 1700–700 cm^−1^, enabling a closer inspection of the phosphate- and carbonate-related bands in terms of position, shape, intensity, and resolution.

The different HAp samples were evaluated by DLS to quantify the size of stable particles in the aqueous suspension prepared for testing (reported in nm). The results are shown in Table 3. Table 4 reports the ζ-potential (mV) measured for the analyzed HAp samples.

### 3.2. In Vitro Test

**Figure 5:**
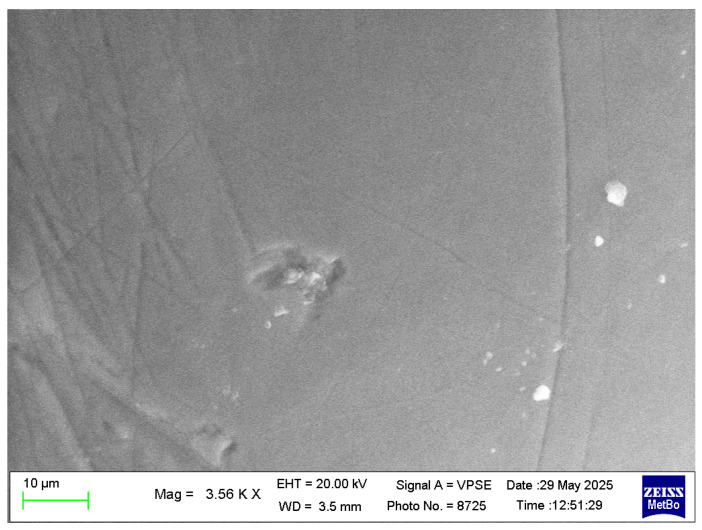
Enamel surface before HAp application, showing wear-related scratches.

**Figure 6:**
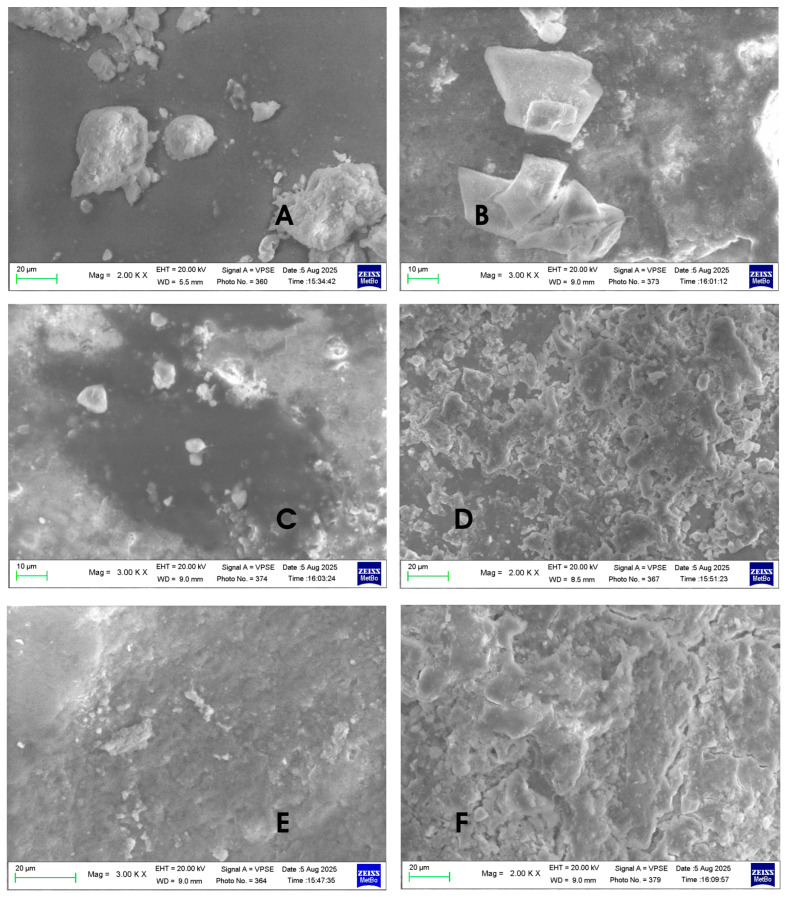
SEM micrographs of the enamel surface after treatment with slurries prepared from (**A**) Kal-HAp; (**B**,**C**) FL-HAp; (**D**,**E**) FL-HAp-SC; and (**F**) microRepair^®^.

**Figure 7:**
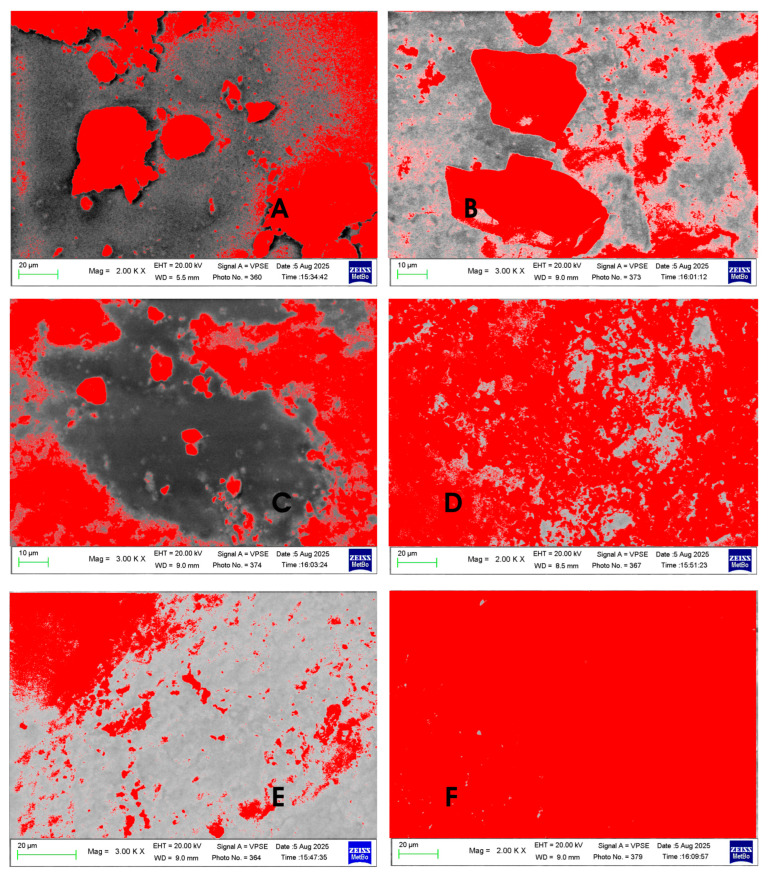
SEM micrographs of enamel surfaces after treatment with slurries prepared from (**A**) Kal-HAp; (**B**,**C**) FL-HAp; (**D**,**E**) FL-HAp-SC; and (**F**) microRepair^®^. Areas covered by hydroxyapatite are shown in red.

As previously described, a slurry was prepared for each HAp by dispersing the material in theappropriate aliquot of artificial saliva, on enamel sections surface (Figure 5) were then brought into contact with the slurry. The objective was to assess enamel coverage after a single application of each inorganic phase. Figure 6 and Figure 7 show that the different HAp samples coat tooth enamel to varying extents. Table 5 reports the percentage of enamel surface covered by each hydroxyapatite, quantified from SEM micrographs using image-analysis software [33,38].

A summary of the data obtained can be found in Table 6.

## 4. Discussion

The objective of this study was to characterize four commercial hydroxyapatites (HAp) in terms of physicochemical properties—size, morphology, and composition—to assess their suitability for oral-care applications. Each HAp was then formulated as a 5% slurry in artificial saliva. Enamel sections were brought into contact with the slurry, and scanning electron microscopy (SEM) was used to evaluate the ability of the inorganic phase to cover the enamel surface after a single application.

Initially, all samples were analyzed by powder X-ray diffraction to determine the crystalline phase. All were found to be hydroxyapatite.

The four HAp samples exhibited diffraction maxima with differing resolutions. Specifically, samples A (Kal-HAp) and D (microRepair^®^, a biomimetic Zn–carbonate-substituted HAp) showed very similar patterns, indicative of medium crystallinity. In these diffractograms, only reflections near 2θ ≈ 31.8°,where the (211) and (112) planes overlap—and at 2θ ≈ 33.0°, assigned to the (300) plane—were clearly discernible; other minor features are not evident, consistent with the moderate crystallinity of these materials.

The XRD patterns of samples B (FL-HAp) and C (FL-HAp-SC) indicate higher crystallinity, as all major reflections are clearly resolved—namely, those at 2θ ≈ 31.8° (assigned to the (211) plane), 2θ ≈ 32.2° (overlapping with the (112) plane), and 2θ ≈ 33.0° (the (300) plane). This sharper, better-resolved peak set is consistent with the greater crystalline order in B and C relative to A and D. The crystallinity values of each HAp, calculated using the Landi method, are reported in Table 1. Samples A and D exhibit a low degree of crystallinity, comparable to that of bone-derived apatite [43,44], whereas samples B and C show higher crystallinity.

The SEM micrographs in Figure 2 reveal distinct morphologies for the four HAp samples. Sample A (Kal-HAp) consists predominantly of flake-like particles withdiameters of approximately 5–20 µm. Sample B (FL-HAp) also shows irregular flakes, with the longer dimension ranging from ~5 µm to >20 µm. Samples C (FL-HAp-SC) and D (microRepair^®^, a biomimetic Zn–carbonate-substituted HAp) exhibited very similar, spongy morphologies composed of micrometer-scale particles. The Ca/P ratios derived from EDS are reported in Table 2.

Infrared analysis (ATR; see Figure 3 and Figure 4) reveals phosphate bands in all samples within the ν_3_(PO_4_) region at ~1037 and ~1100 cm^−1^, reflecting phosphate vibrations in hydroxyapatite. With increasing crystallinity, these components become more clearly resolved, particularly the ~1100 cm^−1^ band. A band near 870 cm^−1^, characteristic of the ν_2_(CO_3_) mode, indicates carbonate substitution within the apatite lattice. Typically, low-crystallinity hydroxyapatites display stronger/broader carbonate features, whereas highly crystalline materials (e.g., heat-treated or high-temperature syntheses) show this band to a much lesser extent.

Consistent with the XRD results, the higher crystallinity in samples B and C and the better-resolved phosphate bands in their ATR-FTIR spectra (Figure 3 and Figure 4) are associated with a lower carbonate content: 2.60% and 2.20%, respectively.

Conversely, samples A and D exhibit lower crystallinity and correspondingly higher carbonate levels: 6.16% and 6.52%, respectively.

Particle-size analysis (DLS) of stably dispersed particles in water highlights two points. First, none of the samples contains free HAp nanoparticles. Second, samples B, C, and D show particles that are sufficiently stable to remain in suspension and be measured, whereas sample A—consistent with its particle size and morphology—did not yield a measurable, stable dispersed fraction in water. As reported in Table 3, the hydrodynamic sizes for B, C, and D fall in the ~1.2–1.6 µm range.

The ζ-potential reflects the electrostatic potential arising from the electrical double layer around particles dispersed in a liquid medium; it governs electrophoretic mobility and colloidal stability. As reported in Table 4, measurements in demineralized water at the same pH yielded negative ζ-potentials with magnitudes between ~14.48 and ~24.92 mV. Because ζ-potential typically spans about −60 to +60 mV and stability increases with the absolute value |ζ|, sample C (FL-HAp-SC; −14.48 mV) shows the lowest magnitude and therefore the least stability in aqueous suspension, whereas samples B (FL-HAp; −24.92 mV) and D (microRepair^®^; −19.25 mV) display greater colloidal stability [45].

After powder characterization, an in vitro assay was performed to assess the ability of hydroxyapatites with differing physicochemical properties to coat tooth enamel. Artificial saliva was prepared according to the literature, and each hydroxyapatite was dispersed at 5% (*w*/*w*) to mimic a plausible oral-care formulation.

Enamel sections were contacted with the slurry for 3 min and then rinsed under running water at room temperature for 10 s.

These conditions were designed to approximate everyday use of an oral-hygiene product (e.g., toothpaste). Figure 5 shows the enamel surface prior to HAp treatment, where typical wear-related scratches from mastication are evident.

Figure 6A–C (Kal-HAp in Figure 6A; FL-HAp in Figure 6B,C) show enamel surfaces with sparse hydroxyapatite particles; in both cases, the particle morphology resembles that of the respective powders. The surfaces in Figure 6D,E (FL-HAp-SC) illustrate patchy, non-uniform coverage: in some areas (Figure 6D), the enamel is moderately and fairly homogeneously coated, whereas in others (Figure 6E), the enamel remains exposed. Finally, Figure 6F (microRepair^®^, biomimetic HAp) shows a continuous uniform coating that obscures the native enamel topography seen in Figure 5. After processing with image-analysis software, the SEM micrographs were color-overlaid (Figure 7), with red highlight regions covered by HAp deposits, and black denotinguncovered enamel.

By colorizing the images, the percentage of enamel coverage achieved by the different hydroxyapatites was quantified.

The data in Table 5 shows heterogeneous, incomplete coverage for A–C, whereas D exhibits high enamel affinity with essentially complete, uniform surface coverage [39].

## 5. Conclusions

This study characterized four hydroxyapatites (HAp) in terms of crystallinity (XRD), morphology (SEM), Ca/P ratio (EDX), carbonate substitution (ATR-FTIR), hydrodynamic size of stable particles (DLS), and colloidal stability (ζ-potential in water). These physicochemical attributes were correlated with the extent of enamel coverage observed in vitro after a single application. Taken together, the data indicate that HAp materials exhibiting moderate-to-low crystallinity, a Ca/P ratio close to that of dental apatite, measurable carbonate substitution, a spongy particle morphology, and sufficiently negative ζ-potential tend to form more continuous, homogeneous coatings on enamel. Among the tested materials, sample D (microRepair^®^, a biomimetic Zn–carbonate-substituted HAp) showed the highest affinity for enamel, achieving near-complete, uniform surface coverage.

These findings suggest practical criteria for selecting or synthesizing HAp intended for oral-care formulations aimed at enamel coating: tuned crystallinity, controlled carbonate substitution, appropriate particle morphology, and colloidal stability. Future work should evaluate coating durability under mechanical challenge (e.g., brushing), adhesion strength, repeated-use cycles, and performance across enamel and dentin substrates, as well as assess clinical relevance.

## Figures and Tables

**Table 1:** Inorganic phase and degree of crystallinity of the HAp samples.

	Inorganic Phase	Degree Cryst.	
Kal-HAp	Hydroxyapatite	16.7	%
FL-HAp	Hydroxyapatite	32.5	%
FL-HAp-SC	Hydroxyapatite	30.3	%
microRepair^®^	Hydroxyapatite	19.8	%

**Table 2:** Ca/P Ratio of HAp used obtained by EDX probe.

	Ca/P Ratio	st. dev.
Kal-HAp	1.55	0.15
FL-HAp	1.61	0.19
FL-HAp-SC	1.69	0.06
microRepair^®^	1.59	0.12

**Table 3:** Dimensions of stable particles of HApdetermined by DLS.

	DLS Dimension	st. dev.	Unit
Kal-HAp	no dimension detected	-	nm
FL-HAp	1299	224	nm
FL-HAp-SC	1696	617	nm
microRepair^®^	1426	464	nm

**Table 4:** ζ-potential of stable particles of HAp.

	ζ-Potential	st. dev.	Unit
Kal-HAp	−17.85	2.07	mV
FL-HAp	−24.92	5.21	mV
FL-HAp-SC	−14.48	0.39	mV
microRepair^®^	−19.25	1.45	mV

**Table 5:** Enamel surface coverage (%) achieved by the hydroxyapatite samples, measured by image analysis of SEM micrographs.

	% Hap Coating	st. dev.	Unit
Kal-HAp	28.83	7.35	%
FL-HAp	31.11	3.12	%
FL-HAp-SC	57.20	33.12	%
microRepair^®^	99.90	0.12	%

**Table 6:** Summary of the data obtained.

	XRD (Inorganic Phase)	Degree of Crystallynity	Ca/P Ratio (EDX)	Morphology (SEM)	Carbonate Substitution	Particle Dimension (DLS)	ζ-Potential	% HAp Coating
Kal-HAp	HAp	16.7%	1.55 ± 0.15	flake-like particles	6.16%	-	−17.85 ± 2.07 mV	28.83 ± 7.35%
FL HAp	HAp	32.5%	1.61 ± 0.19	flake-like particles	2.60%	1299 ± 224 nm	−24.92 ± 5.21 mV	31.11 ± 3.12%
FL HAp SC	HAp	30.3%	1.69 ± 0.06	spongy	2.20%	1696 ± 617 nm	−14.48 ± 0.39 mV	57.20 ± 33.12%
microRepair^®^	HAp	19.8%	1.59 ± 0.12	spongy	6.52%	1426 ± 464 nm	−19.25 ± 1.45 mV	99.90 ± 0.12%

## Data Availability

The original contributions presented in this study are included in the article. Further inquiries can be directed to the corresponding author.
